# 320 × 240 SPAD Direct Time-of-Flight Image Sensor and Camera Based on In-Pixel Correlation and Switched-Capacitor Averaging

**DOI:** 10.3390/s25216772

**Published:** 2025-11-05

**Authors:** Maarten Kuijk, Ayman Morsy, Thomas Lapauw, Thomas Van den Dries, Wannes Nevens, Mohamed A. Bounouar, Hans Ingelberts, Daniel Van Nieuwenhove

**Affiliations:** 1 ETRO.RDI, Vrije Universiteit Brussel, Pleinlaan 2, 1050 Brussels, Belgium; ayman.morsy@vub.be (A.M.); thomas.lapauw@vub.be (T.L.); thomas.van.den.dries@vub.be (T.V.d.D.); wannes.willem.nevens@vub.be (W.N.); hans.ingelberts@vub.be (H.I.); 2 SONY Depthsensing Solutions, Rue Jules Cockx 8, 1160 Brussel, Belgium; mohamed.a.bounouar@sony.com (M.A.B.); daniel.vannieuwenhove@sony.com (D.V.N.)

**Keywords:** 3D-ToF, CA-dToF, LIDAR, switched capacitors, exponential moving average, SPAD, depth sensing

## Abstract

Correlation-Assisted Direct Time-of-Flight (CA-dToF) is demonstrated for the first time on a large 320 × 240-pixel SPAD array sensor that includes on-chip high-speed timing support circuitry. SPAD events are processed in-pixel, avoiding data communication over the array and/or storage bottlenecks. This is accomplished by sampling two orthogonal triangle waves that are synchronized with short light pulses illuminating the scene. Using small switched-capacitor circuits, exponential moving averaging (EMA) is applied to the sampled voltages, delivering two analog voltages (VQ2, VI2). These contain the phase delay, or the time of flight between the light pulse and photon’s time of arrival (ToA). Uncorrelated ambient photons and dark counts are averaged out, leaving only their associated shot noise impacting the phase precision. The QVGA camera allows for capturing depth-sense images with sub-cm precision over a 6 m range of detection, even with a small PDE of 0.7% at an 850 nm wavelength.

## 1. Introduction

Time-of-flight (ToF) imaging has emerged as a key technique for depth sensing, with growing attention due to its potential in applications requiring efficient power usage, high-resolution imaging, and accurate distance measurements with low uncertainty [1]. These performance demands are particularly relevant in areas such as automotive, machine vision, and portable consumer devices, including smartphones and laptops [2]. Among ToF approaches, the two dominant methods are direct time-of-flight (dToF) and indirect time-of-flight (iToF).

In iToF, a modulated light source illuminates the scene in synchronization with a photonic mixer device shutter, and the ToF is extracted from the phase shift between the emitted and reflected signals. There are two main operation modes of the used light source in iToF: either pulsed emission or continuous-wave modulation, typically with sinusoidal or square waveforms [3]. Photonic mixer devices are typically CMOS sensors designed to modulate the electric field within the sensor. The electric field modulates the generated photocurrent to multiple nodes, synchronized with the light source [4,5]. Among the various iToF techniques, the amplitude-modulated continuous-wave (AMCW) approach is particularly prevalent due to its simple implementation and high robustness [4]. In this configuration, phase information is extracted by sampling the incoming light with four gates corresponding to a 50% duty cycle-modulated light signal. Despite its advantages, the performance of AMCW systems can be adversely affected by shot noise induced by ambient illumination, which limits operational depth accuracy. As a result, previous studies have reported that achieving VGA or higher pixel resolutions typically restricts the operational range to approximately four meters under strong ambient lighting and high modulation frequencies [6,7,8]. Furthermore, when the irradiance is high, the finite charge capacity of small pixel pitches can lead to pixel saturation. To mitigate this effect, pixel binning techniques have been proposed as an effective solution [6,9].

In contrast to iToF, dToF measures photon arrival times directly, generating a histogram of detected events. This approach employs short-pulsed laser illumination and avalanche detectors, with arrival times digitized or converted into analog values [3]. The digitized output of arrival time is a popular implementation in dToF with several architectures, including ring oscillators [10], multiphase clocking schemes [11], and pixel-level Vernier delay lines [12]. Ongoing advancements in TDC circuit design and calibration methods continue to enhance linearity performance [13]. In dToF operation, multiple laser return pulses are accumulated across several cycles, forming a histogram where each bin corresponds to a discretized time interval determined by the TDC resolution and system detection range. Ambient light contributes a relatively uniform background level across the histogram, superimposed with random variations due to shot noise, while the true laser return signal appears as peaks with higher counts.

Single-photon avalanche diode (SPAD)-based dToF imagers are widely adopted because SPADs offer a high sensitivity and single-photon detection capability, enabling a performance ranging from a few meters up to several kilometers, depending on the laser power. However, the precision and accuracy of these systems are strongly influenced by the TDC architecture and its timing resolution, which, in turn, affect the data bandwidth of the sensor. Depending on the pixel count and frame rate, the resulting on-chip data throughput can span from gigabits to terabits per second. As resolution increases, the data bandwidth rises accordingly, leading to greater power consumption and restricting the achievable frame rate. This imposes a substantial burden on data management and system integration complexity.

To address this challenge, various techniques have been introduced to perform partial data processing directly on-chip, thereby reducing the data volume that must be transferred off-chip. One approach involves integrating histogram computation within each pixel, allowing peak detection to occur locally after compensating for background noise [14]. However, this implementation, fabricated in a 40 nm front-side illumination (FSI) technology, results in a pixel size of 114 × 54 μm^2^, which poses limitations for scaling to high-resolution arrays. Another significant challenge in dToF pixels is pile-up distortion, caused by SPAD dead time and limited TDC processing speed, which can lead to errors in identifying the correct return peak [15].

There have been efforts in the last few years to overcome the fundamental challenges in iToF and dToF technologies, such as SPAD-based iToF and hybrid ToF [16,17,18,19]. Among these efforts is the recent development of a Correlation-Assisted Direct Time-of-Flight pixel (CA-dToF), which was first presented based on sine and cosine demodulation using a single-stage exponential moving average (EMA) for measuring depth, using a 32 × 32 prototype array [20]. Furthermore, an analytical model was developed, and a 100 klux solar ambient light suppression was demonstrated [21]. The pixel showed reliable performance under challenging ambient light conditions, and flexibility in performance and optimization. The systematic and environmental challenges of the pixel implementation showed common challenges to iToF, such as phase rapping, multipath interference, and source follower mismatch [4]. However, the CA-dToF pixel can tackle such challenges, as presented in [22].

We now demonstrate the operation of a camera containing a QVGA 320 × 240-pixel image sensor whereby the pixels use a cascade of two averaging stages, increasing the in-pixel level of available averaging (AA) to the product of the two. The first averaging stage samples at each SPAD event; the second stage is this time operated at a switching rate *which is varied during each sub-frame*, for a quick convergence of the average voltages and optimal noise reduction.

If the number of incoming photon events within a subframe is less than the AA, we obtain unharmful over-averaging, assuming uniformly spread ambient (A) and reflected signal (S) photons. For this over-averaging situation, a comprehensive model will now provide an estimation of the depth’s standard deviation.

Furthermore, in this work, we demonstrate the use of triangular signals (TSIN, TCOS) instead of sinusoidal signals, simplifying the analog driving circuit. An analog photon counter is also implemented for gray-image capture. The pixel has an area of 30 × 30 μm^2^, including SPAD (Ø = 10 μm), delivering a small fill factor (8.7%) and hence low PDE (~0.7%) at l = 850 nm.

## 2. Used Subcircuits Based on Switched Capacitors

Three different circuits with common circuitry elements and operating principles are discussed first: two for averaging voltage samples and one for photon counting. The latter is included for acquiring a gray image with and without laser illumination of the scene, which is required to validate the provided depth precision model.

### 2.1. Exponential Moving Average and Its Implementation

When dealing with a noisy and continuous stream of measurements of a given quantity, predicting the underlying trend based on previous observations presents a significant challenge.

**Figure 1:**
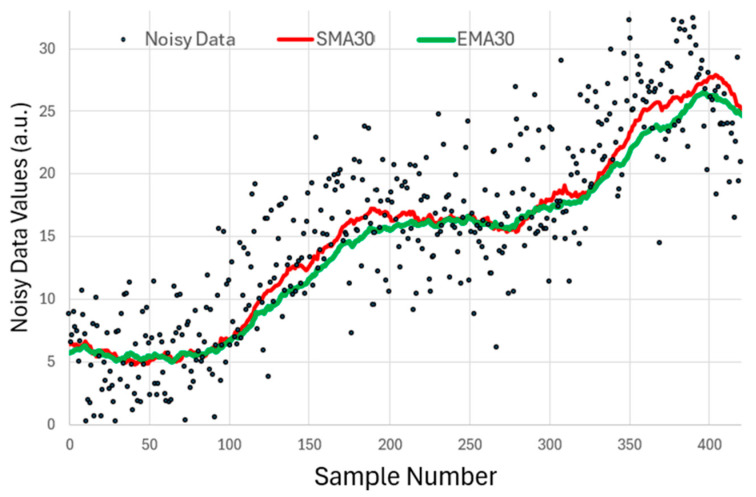
An example of a noisy data stream on which the simple moving average (SMA) and the exponential moving average (EMA) are applied with $n_{avg}$ = 30. A similar averaging is obtained.

A straightforward approach is the simple moving average (SMA), in which each predicted value is obtained by computing the mean of the most recent $n_{avg}\;$ samples. Based on a Gaussian spread assumption, noise is reduced with $1⁄\surd n_{avg}$ . However, if the underlying trend is changing, one must compromise between accuracy and precision depending on the number of samples. Figure 1 shows this SMA, over 30 samples, updated after every incoming sample and applied on a sequence of values with high noise content.

Another type of averaging is the exponential moving average (EMA). In this system, one does not need to memorize the list of the last $n_{avg}\;$ samples; one only keeps the latest average itself. Every time a new sample enters, an update to this average is made by taking the new sample into account in a weighted way depending on an averaging number, n_avg_: this parameter also reflects (roughly) over how many samples averaging is performed.

**Figure 2:**
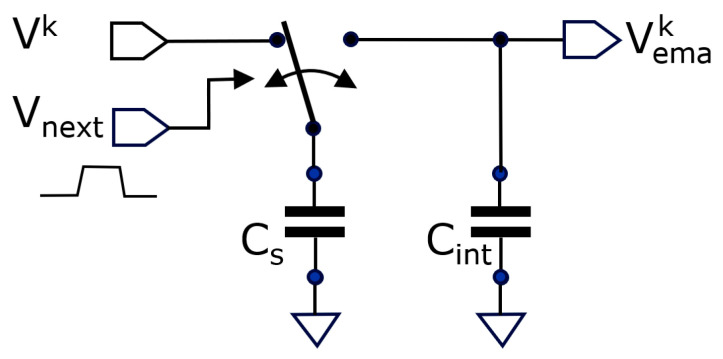
This circuit implements the EMA: the capacitor C_s_ charges to $V^{k}$ ; when a pulse is applied to V_next_, the switch toggles to the right, during which C_s_ and C_int_ become shorted. A new voltage based on charge sharing is established, resulting in the updated V_EMA_.

These SMA and EMA averages $V_{SMA}^{k}\;and\;V_{EMA}^{k}$ are calculated as follows:

(1) $$V_{SMA}^{k}=\frac{1}{n_{avg}}\sum_{i=k-\left(n_{avg}-1\right)}^{k}V^{k},$$

(2) $$V_{EMA}^{k}={\frac{\left(n_{avg}-1\right)}{n_{avg}}V}_{EMA}^{k-1}+\frac{1}{n_{avg}}.V^{k},$$

whereby $V^{k}\;$ represents every new incoming sample. Notice that the samples may arrive at irregular intervals. The average updates at the arrival of each new sample. Besides not needing to memorize a table of samples, the EMA has the property to weigh the importance of the past samples in a variable way; the longer ago a sample was taken, the less it weighs into the average [23]. Depending on the application, this can be considered an advantage or a disadvantage. However, for image sensors, the EMA principle has the undeniable advantage that it only needs to memorize one value. Moreover, it can be implemented with the elementary circuit given in Figure 2.

From charge conservation (before and after short circuiting), we obtain the updated EMA as follows:

$$V^{k}.C_{s}+V_{EMA}^{k-1}.C_{int}=V_{EMA}^{k}\left(C_{s}+C_{int}\right),$$

(3) $$\to \;V_{EMA}^{k}=\frac{\left(n_{avg}-1\right)}{n_{avg}}V_{EMA}^{k-1}+\frac{1}{n_{avg}}.V^{k},$$

(4) $$n_{avg}=1+\left(\frac{C_{int}}{C_{s}}\right),$$

whereby Equation (3) is the same as Equation (2), with the averaging number $n_{avg}\;$ depending on the ratio of the two involved capacitors. These equations prove that this simple circuit can implement the EMA based on voltage domain signals.

**Figure 3:**
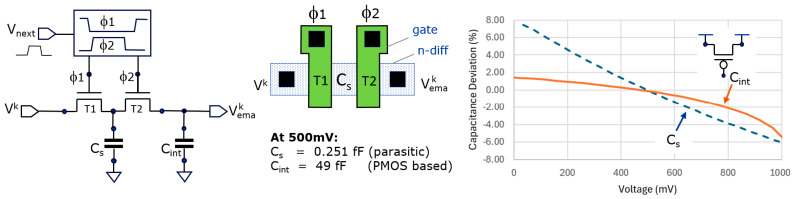
The practical EMA implementation consists of generating non-overlapping clocks (f_1_ and f_2_) in response to an edge transition from V_next_, driving the gates of two NMOS transistors (left); the parasitic capacitance of the substrate diffusion diode between the two transistors forms C_s_ (center); the non-linear behavior of C_s_ and C_int_ form spice simulation and are shown on the right.

The practical implementation in CMOS circuitry may use various types of capacitors and switches. CMOS switches (using both an NMOS and a PMOS transistor) as well as pass gates (based solely on an NMOS or PMOS transistor) can be used. We opted to use NMOS transistor pass gates, allowing the construction of a very low parasitic capacitance C_s_. Figure 3 shows how such a small C_s_ can be made: we use the parasitic drain/source between T1 and T2 to obtain the smallest achievable area depending on design rules. The capacitance is derived from the parasitic extract and is 0.251 fF. C_int_ is preferably made using this technology with a PMOS transistor. We use here one that has a gate capacitance of 49 fF, leading to an $n_{avg}$ of ~200. These capacitances show, unfortunately, a non-linear behavior (Figure 3, right) that can result in accuracy errors (further discussed in Section 3.3.3). However, in practice, their non-linearities are largely canceled by using differential and quad-phase measurements.

There is further some variability in C_s_ when based on a parasitic node for its implementation, due to process variation and statistical spread. Hence the implemented value for $n_{avg}$ also varies. When used merely for averaging, this is of a lesser concern than when used for photon counting, where the gain of the counter becomes variable too.

The clocks (F1, F2) must not be high simultaneously to avoid a shorting of the two capacitors; this can easily be achieved with a few logic gates, e.g., two NOR gates and a NOT gate. A relevant concern when switching capacitors is the effect of the kTC noise. At room temperature, it is 4 mV for C_s_ and 0.3 mV for C_int_. The impact of the 4 mV is reduced by a factor 200 when the two caps are shortened, so the 0.3 mV on the output C_int_ prevails. This value is smaller than the smallest ADC step of 0.5 mV (when operating the ADC at 11-bit resolution over a 1 V span) and is therefore neglected [22].

### 2.2. More Averaging: Use of Switched-Capacitor Low-Pass Filter(s)

**Figure 4:**
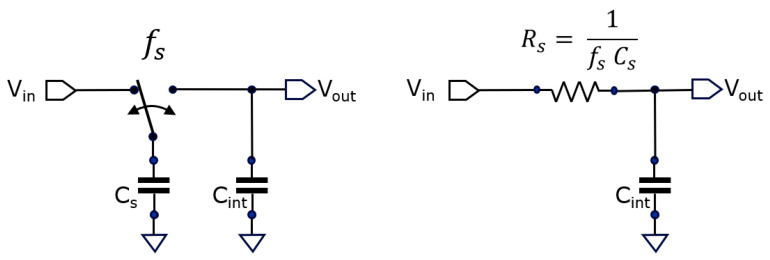
By periodically toggling the switch, a low-pass filter is constructed, with −3 dB corner frequency settable by the toggling frequency $f_{s}$ .

When one needs much larger averaging numbers, just making an extremely large C_int_ is not an option inside a small sensor pixel. Alternatively, a second averaging stage can be implemented based on a switched-capacitor circuit using a similar circuit topology (Figure 4), based on a textbook low-pass filter circuit.

The corner frequency $f_{-3dB}$ of this low-pass filter is given by the following:

(5) $$f_{-3dB}=\frac{1}{2\pi R_{s}C_{int}}=\frac{f_{s}.C_{s}}{2\pi C_{int}},$$

whereby $f_{s}\;$ represents the switching frequency of the switch. Notice that this circuit is the same as the EMA stage. However, this time, a regular switching is applied instead of sampling at the occurrence of each new sample. Similarly, an averaging number, $n_{avg}$ , can be attributed to this switched-capacitor low-pass filter based on Equation (4). A nicety of this type of low-pass filtering is the fact that its corner frequency $f_{-3dB}\;$ can be varied in time by varying the switching frequency $f_{s}$ , something we use for optimizing camera operation further on.

### 2.3. Analog Counter for Counting Photons

**Figure 5:**
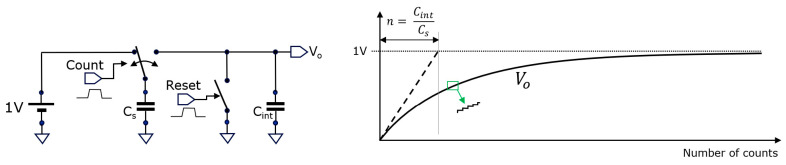
An analog counter based on a switched-capacitor principle, useful for counting events like incident photons. When zooming-in, the step-like behavior becomes visible (in green).

Another textbook circuit is the analog counter, similarly based on the switched-capacitor operation shown in Figure 5.

In this case, a pulse on the Reset input resets the voltage on the capacitor C_int_ to 0 V at the start of the counting process. At each count event, the switch is toggled. *V_o_* shows an exponential step-like voltage increase over the number of counts; by using the tangent at the start of the slope, the value of C_int_/C_s_ can be constructed graphically. This is similar to finding the RC-time constant in the case of charging a capacitor C through a resistor R.

## 3. Correlation-Assisted Direct Time-of-Flight (CA-dToF) Principle

In this section, we assume that the AA is much greater than the number of photon events (A + S) detected during a subframe, which is also the case for the indoor experiments of the next section. This assumption has the advantage that a simple comprehensive analytical model can be applied for estimating the precision of the distance measurements. First, the operating principle is explained, and then the accuracy and precision of the depth sensing method are discussed.

### 3.1. Operating Principle

To achieve a high averaging level, we use a cascade of two averaging stages. The first averaging stage samples at each SPAD event (with n_avg1_ = 200); the second is operated as a low-pass filter with a variable switching frequency and with n_avg2_ = 400. These two averaging numbers are used for the simulations below and measurements further on. This brings the in-pixel AA to the product of the two: AA = n_avg1_ × n_avg2_ = 8 × 10^4^.

In the example case below, the number of incoming events within a subframe is less than the AA, and we assume that we obtain unharmful *over-averaging* because the number of ambient photons per subframe (A) and that of signal photons (S) are spread uniformly enough.

**Figure 6:**
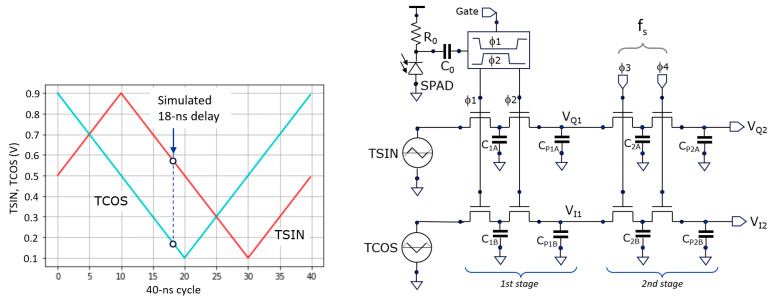
The correlation functions TCOS and TSIN (**left**) and the schematic (**right**) of the two-stage averaging system for correlating the incident ToAs of photons with these functions.

Figure 6 shows the two correlation voltage waves (left) generated in the camera. Instead of orthogonal sine and cosine waves for correlation, we use the shown triangle waves (TCOS and TSIN). These are also orthogonal but have the advantage that they can be generated on-chip using simple current sources.

The voltages oscillate between 100 mV and 900 mV, a range in which NMOS transistors (in a 180 nm CMOS technology) operate as pass-gate switches. Assuming 500 mV as the center voltage, the estimated distance can be calculated as follows:

(6) $$D=\frac{\Gamma }{4}\;\left[1+\frac{\left(0.5-\;V_{I2}\right)}{\left|V_{Q2}-0.5\right|+\left|V_{I2}-0.5\right|}\right]\;\;when\;\left(V_{Q2}-0.5\right)>0\;,$$

(7) $$D=\frac{\Gamma }{4}\;\left[3+\frac{\left(\;V_{I2}-0.5\right)}{\left|V_{Q2}-0.5\right|+\left|V_{I2}-0.5\right|}\right]\;\;when\;\left(V_{Q2}-0.5\right)<0\;,$$

where Γ is the unambiguous distance (=T_cycle_ × c/2), which is 6 m in this cycle period (T_cycle_ = 40 ns). When using the sine and cosine wave, the arctangent function would appear in the depth estimation.

**Figure 7:**
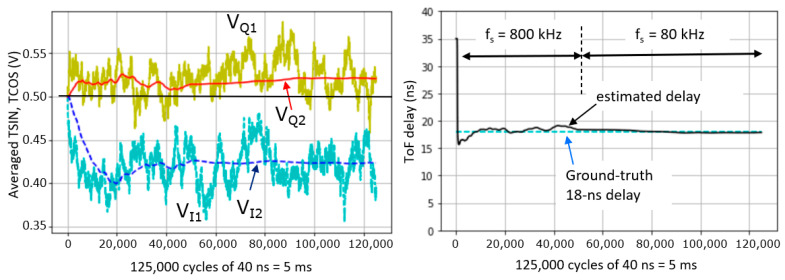
Statistical simulation of a frame of 125 k cycles showing the averaging of the sampled TCOS and TSIN after the first and after the second averaging stages (left). On the right, the distance is calculated based on Equations (6) and (7) and the indicated toggling frequency f_s_ is reduced by a factor of 10 after 40% of the frame.

In the simulation of Figure 7, there are 125 k cycles of 40 ns; from the laser pulse that is emitted at the onset of each started cycle, only *one signal photon* (on average) returns after 18 ns from the scene *for every 100 cycles*, and *one ambient photon* (on average) *for every 25 cycles,* following the Poisson distribution. Per subframe, S = 1250 (on average) and A = 5000 (on average). The ambient-to-signal ratio (ASR) equals A/S = 4. Left in the figure, the signals V_Q1_ and V_I1_ are updated at each incoming event, originating from both A and S photons. Because the number of ambient events is approximately four times greater than signal events and randomly occurring within T_cycle_, the signals exhibit significant noise. On average, the ambient events pull the voltage toward the 500 mV midpoint. In contrast, one out of every five events are signal photons, located at 18 ns within T_cycle_, as indicated in Figure 6 (left). Each such signal event pulls V_Q1_ toward 580 mV and V_I1_ toward 180 mV. In the absence of ambient light (A = 0), V_Q1_ would stabilize at 580 mV and V_I1_ at 180 mV, yielding a total amplitude of 400 mV. However, because ambient events occur four times more frequently and tend to shift the signals toward the 500 mV midpoint, the contribution of the signal events is effectively reduced by a factor of 5, similar to a ratio division with resistors. As a result, V_Q1_ fluctuates around 516 mV, V_I1_ around 436 mV, and the overall amplitude is reduced to 80 mV, i.e., by a factor of $1+\frac{A}{S}=5$ . To better recover these voltages, one can look to V_Q2_ and V_I2_, which effectively average out the noise but leave the 80 mV signal intact.

This amplitude reduction is an inherent part of the charge-sharing mechanism in the first stage. The average voltages deliver a depth estimate. The amplitude $(\left|V_{Q2}-0.5\right|+\left|V_{I2}-0.5\right|)\;$ gives an idea of the ASR. Note that a very large ASR can reduce the amplitude so much that the quantizing of the subsequent ADC becomes a limiting factor. But, as long as that is not the case, a large variety of values for A and S can be present in the scene, while the amplitude of the signal still provides a good indication as to how meaningful the signal is; it can thus serve as a confidence measure.

Very conveniently, the averaging system can never saturate and, as such, increasing the exposure time is never harmful. This is a key advantage of averaging compared to integrating, and this fact is demonstrated further on in the measurements showing a range of ~3 orders of magnitude to choose the exposure time without running into saturation (Section 5.5). Additionally, Section 5.6 demonstrates a variation in ASR from 0.2 to 66.

Concerning the choice of the low-pass corner frequency $f_{-3dB}$ : the lower we choose this frequency, the better the precision will be. However, taking this frequency too low generates a considerable spillover from the previous measurement frames, which is detrimental to the measurements’ accuracy. When performing single sub-frame imaging, only a less harmful distance image lag occurs. It is, however, the intention to achieve frame-independent measurements. With switching frequency f_s_ = 80 kHz and $n_{avg2}$ of 400, one RC-time will be $n_{avg2}$ /f_s_ = 5 ms.

To avoid waiting for five RC-time periods (25 ms) for convergence, and to achieve an accuracy better than 1%, we choose to speed f_s_ up during the first 40% of the exposure period by setting the switching rate to 800 kHz. This is enough to accelerate convergence and to obtain V_Q2_ and V_I2_ to their approximate end-values quickly, leaving time for the remaining 60% of the period to average out the noise, strongly improving precision.

In Figure 7, one can observe that, during the first 40% of the period, V_Q2_ and V_I2_ strongly try to catch up with the changes in the (noisy) first averages V_Q1_ and V_I1_. And, when the switching frequency is reduced to 80 kHz, their updates are much more conservative. In the indicated estimated (phase) delay at Figure 7 (right), first a quick “reset” towards the 18 ns ground truth occurs. Then, smaller adjustments in the direction of the ground truth happen, improving both the accuracy and precision.

### 3.2. Depth Precision Model

The analytical model discussed in the previous publications [20,21,22] only considered the limited averaging capability in-pixel, leading to a relatively complex expression for the distance standard deviation (STDV), or depth precision. A comprehensive analytical model can be derived, since large averaging can now be achieved in-pixel.

Specifically, we consider the case in which the total available averaging exceeds the number of photon triggers within a measurement subframe (AA > A + S). Under this assumption, a simplified analytical model is derived by treating the averaging as effectively complete. This assumption is justified because the incident photons are statistically distributed across the measurement subframe, ensuring that both ambient (A) and signal (S) events contribute to the averaging process. Such full averaging is, in fact, realized with our camera in indoor scenarios presented in the following sections.

#### 3.2.1. Use of Multiple Subframes

In practical cases, *multiple subframes* are captured to improve depth sensing quality. Therefore, the *total* number of signal and ambient numbers, $S_{t}\;$ and $A_{t}$ , play a role in determining the depth precision:

(8) $$S_{t}=n_{sf}.S\;,$$

(9) $$A_{t}=n_{sf}.A\;,$$

whereby S  and A  are the average number of signal and ambient photons in *a single subframe* and $n_{sf}\;$ constitutes the number of subframes that play a role in the averaging.

For instance, in the case of a differential measurement, $n_{sf}\;$ = 2; when four phases are employed, $n_{sf}\;$ = 4. Furthermore, if neighboring pixels are considered for spatial filtering, this number increases further (by ×4 in the one proposed in Section 5.3).

#### 3.2.2. Effect of Laser Pulse, SPAD Timing Jitter, and System Jitter

Received signal photons (S) are spread in accordance with the emitted laser pulse width. A second source that spreads the ToA is the SPAD’s timing jitter $\sigma_{SPAD}$ . And more jitters originating from the system can be grouped in a system jitter, $\sigma_{Sys}.$ These all add up based on RMS addition. The total will be reduced based on the total number of signal photons involved as follows:

(10) $$\delta_{laser+SPAD+Sys}=\frac{c}{2}.\frac{\sqrt{\sigma_{laser}^{2}+\sigma_{SPAD}^{2}+\sigma_{Sys}^{2}}}{\sqrt{S_{t}}},$$

whereby $\sigma_{laser}$ is half the laser pulse width. In our system described below, the laser pulse width is the greatest source of timing jitters, though only a few photons are needed to reduce the effect of the laser pulse width to below one percent. Consider a commercially available VCSEL array generating 2 ns wide pulses to illuminate the scene; then, 25 signal photons reduce the jitter from 1 ns down to 200 ps jitter, corresponding to 3 cm variation, or 0.5% of an unambiguous distance of 6 m. The assumption that the laser pulse has a Gaussian shape is typically not met; in our experiments, it has more of a top-hat shape. Still, the narrowing of the time/distance spread is somewhat in accordance with Equation (10).

The received signal photons (S) also induce shot noise, which generates noise on the two average voltages. However, if these are sampled simultaneously (like in Figure 6), these noise components are correlated and their effect on the distance is canceled or strongly reduced; therefore, we consider this effect as second order.

#### 3.2.3. Effect of Ambient Light

Ambient light photons, $A_{t}$ , are an important factor in the noise that leads to the distance STDV. The signal-to-(uncorrelated)-noise ratio is as follows:

(11) SNR=StAt3 ,

where the *divide by 3* comes from the *effective value* that a triangle wave generates when averaging according to the root-mean-square (RMS) principle. When using sine and cosine waves, the divisor would be 2. From the partial derivative of the distance one can deduce the following:

(12) $$\delta_{ambient}=\frac{\Gamma }{4}\frac{1}{SNR}\;,$$

or, by substituting Equation (11) into Equation (12) one obtains the following:

(13) δambient= Γ4×At3St .

The total STDV on the distance is calculated using the RMS method:

(14) $$\delta_{total}=\sqrt{{\delta_{ambient}}^{2}+{\delta_{laser+SPAD+Sys}}^{2}}\;.$$

In case there is little ambient light present, the effect of the laser pulse width will dominate. A further refinement of these calculations shows that deviations occur in the triangle wave corner cases (at 0, 10, 20, and 30 ns in Figure 6, left) due to rectifying effects. These effects are relatively small when the laser pulse width is small compared to the cycle time; as such, we consider it a second-order effect.

### 3.3. Accuracy Considerations

The accuracy of depth measurements can be affected by several factors. Imperfections in SPADs such as *dark counts*, *afterpulsing*, and *deadtime* are some of their inherent limitations affecting the measured distance accuracy and precision. In addition, non-idealities in the VCSEL array and its driver, as well as the presence of multiple optical propagation paths, introduce further sources of error that degrade measurement accuracy. In this section, we address these challenges and their effect on CA-dToF.

#### 3.3.1. Effect of Laser Pulse Shape

The shape of a commercial VCSEL array pulse is closer to a “top-hat” than to a Gaussian pulse. CA-dToF estimates the position based on the center of mass (CoM) of the laser pulse in the time domain. When the light source changes temperature or ages, the laser light output pulse distribution may change, leading to a CoM deviation that can be regarded as a general distance offset for the full array.

Furthermore, in the triangle wave corners, some level of rectification occurs, leading to a deviation from the real CoM, generating a *cyclic error*. This cyclic error is also in proportion to the laser’s pulse width. To gauge the importance of this error, we further characterized it with the camera demonstrator (Section 5.4).

#### 3.3.2. Effect of SPAD’s Non-Idealities

A first element to consider is the SPAD’s *dark count rate* (DCR). DCR events are uncorrelated with the laser pulse emission and thus behave similarly to events from ambient light photons. Some SPADs may exhibit a significant high DCR, and, as with very high ambient photon rates, this is discoverable by observing the amplitude of the averaged signal. When using spatial filtering, a singular *high-DCR SPAD* is seamlessly included *with less impact* on the estimated distance outcome (Section 5.3).

*Afterpulsing* is correlated with delayed events from the received laser pulse, leading to accuracy errors. This must be avoided by any available means. The likelihood and delay of the afterpulsing will influence its impact.

The SPAD’s *deadtime* can introduce two major effects, as described in more detail in [22]. The first one is the shadowing of the ambient photons: if S signal events happen often, e.g., every Tcycle, then, during the subsequent deadtime, ambient photon events are suppressed; thus, the assumption of the equal spread of ambient events is no longer valid. Similarly, the deadtime can also obstruct the triggering from signal photons arriving quite immediately after a previous signal photon event. This is referred to as a pile-up. In both cases, objects appear closer than the ground truth, leading to an accuracy error. If these issues become relatively important for an application, an approach can be taken whereby multiple SPADs are dedicated to serving one pixel, mitigating the deadtime effects, e.g., 2 × 2 or 3 × 3 SPAD cluster topologies can be implemented. In this case, each SPAD can have its own non-overlapping clock generation, operating independently, yet they can all average out on the same C_int_. In the camera demonstrator, the number of photons received per cycle is limited so that deadtime effects can be considered negligible.

#### 3.3.3. Errors Due to Non-Linear Capacitor Behavior

As mentioned in Figure 3, it is possible that C_s_ and C_int_ show non-linear behavior depending on the particular implementation. The non-linear effect on C_int_, according to our analysis, is not important. Additionally, in our implementation, the variation in +1% or −1%, on its own, is relatively small.

However, in our implementation, the non-linearity (−6% to +6) of C_s_ is of larger concern. One way to mitigate this variation is to make it less dependent on a junction capacitance by complementing it with an additional linear capacitor. However, this would inevitably make C_s_ much larger and thus lower the n_avg1_ of the first stage significantly. So, we choose not to do so. Consequently, when experimentally evaluating the system based on a single subframe, we encountered a serious cyclic error which was *amongst others* due to this non-linear C_s_. However, by operating differentially, i.e., with two-phased operations, the differential amplitude has at one side an increase in amplitude, and on the other side a decrease in amplitude, *largely canceling each other out*. We assume that this works out this way thanks to the fact that the curve C_s_ in Figure 3, representing the deviation of the nominal capacitance value, is close to a straight line. The special Section 5.4 also measures the cyclic error in the case of differential operation and demonstrates that the cyclic errors remain indeed small enough, underpinning this statement based on measurements. The use of quad-phase operations (in the six-subframe case) is even better in this respect.

#### 3.3.4. Multipath Errors

Multipath is an important element in systems that work on phase estimation. When there are multiple ways in which the pulsed light can travel and reach a pixel, complex vectors add up, and the phase of the vector sum generates the estimate of the distance. The larger the contribution of the interferer to the victim, the more the distance outcome deviates from the real distance. This crosstalk is more detrimental when the victim has a small amplitude, as with distant and/or low-reflective objects. The interfering photons may follow different paths, such as (i) when having double reflections in the corner of a room, (ii) when having reflections on a semi-transparent object, like a glass door, or a glass bottle, or (iii) when having light received from both an edge of an object and, e.g., a more distant wall. The last condition can, however, be treated further on in the image processing chain. Multipath problems are known in iToF systems. Likewise, for CA-dToF, they limit the depth accuracy.

## 4. Camera Demonstrator

In this section, the hardware is described that was developed to demonstrate the operation of CA-dToF on a large image sensor. A custom chip was designed in 180 nm high-voltage CMOS technology (from X-Fab), utilizing foundry-validated SPADs. Testability was a main design target, with internal option bits allowing for many ways of sensor operation. First, the pixel will be described, followed by a view of the overall camera demonstrator.

### 4.1. Pixel Circuitry

**Figure 8:**
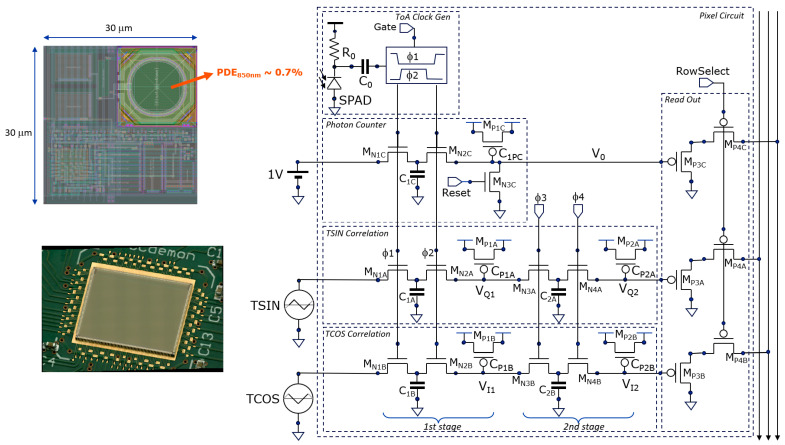
The pixel circuit has a non-overlapping clock generator, a photon counter, and two-stage averaging for the sampled triangular TSIN and TCOS signals.

A single SPAD per pixel has been implemented. It is passively quenched, with an R_0_ of 350 kW, followed by two capacitors (28 fF) in series to handle the high voltage (C_0_ = 14 fF). This AC-coupling was the only option for making an array-type demonstrator in the given technology. At the ToA, two non-overlapping clocks (F1, F2) are generated for toggling the photon counter stage and the TSIN and TCOS first averaging stages simultaneously, as shown in Figure 8. The second stage averaging filters have their non-overlapping clocks (F3, F4) switched with a settable frequency, like in the simulation (Section 3.1).

The analog photon counter is available for gray-image capture and depth-model verification. Its voltage V_0_ goes exponentially towards 1 V (Fgure 4); however, we limited its use up to 2/3 of the maximum 1 V because the count fidelity worsens close to the asymptotic 1 V level. At 2/3 is when ~1715 photons have been counted, which we define as the counter’s full depth.

Furthermore, we use the photon statistics to calibrate the conversion factor between the ADC’s digital number and the incident number of photons, knowing that we operate in a shot-noise-limited regime. There is a measured statistical spread of ~4% over the counter gain of the pixels due to the statistical variation on the parasitic C_s_ in the photon counters.

For a simple on-chip implementation (Figure 7), triangular signals (TSIN, TCOS) are applied instead of the sinusoidal signals. A nanosecond-window gate is present that allows the system to operate as a high-speed time-gated fluorescence lifetime camera [24], not discussed here. The total pixel area is 30 × 30 μm^2^ including SPAD (Ø = 10 μm), delivering a fill factor of 8.7% and hence a low PDE of ~0.7% at l = 850 nm. The readout is performed through PMOS voltage followers with PMOS select switches.

### 4.2. Camera and Image Sensor Circuitry

The setup was developed as a proof-of-concept for the proposed averaging principles and triangle waveform generation, offering a high degree of configurability. For example, the column ADC can be configured for 8-, 9-, 10-, or 11-bit resolution, and its conversion slope can be selected to occur in steps of 50, 100, and 200 MHz. Many of the internal signals, including the triangle wave voltages, can be verified on the oscilloscope via the internal multiplexers connected to test pads.

**Figure 9:**
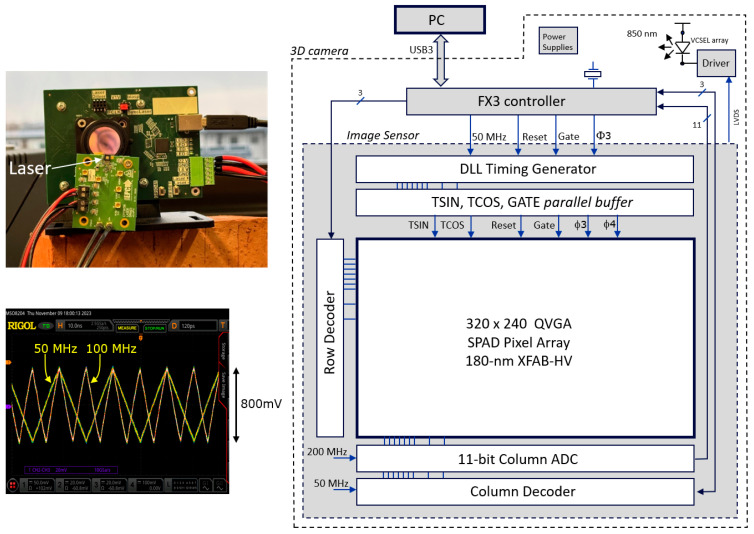
The camera is controlled by a PC over USB3 (right). An FX3 microcontroller (Infineon) provides communication with the PC through direct memory access with the image sensor chip. Left: experimental camera setup and measured triangular waveforms generated on-chip.

Figure 9 (right) shows the camera architecture together with the image sensor circuit. The FX3 microcontroller forms the interface between the PC and the image sensor to transfer the image data over USB3.0 and to provide clock and measurement arbitration signals to the sensor. The FX3 does not perform image data manipulation; this is performed on the PC.

From the 50 MHz clock provided by the microcontroller, the image sensor generates a 40 ns cycle (Tcycle), and, through a current-starved delay line, 64 subdivisions of 625 ps periods are defined. A delay-locked loop regulates the current so that these 64 steps align with the 40 ns cycle. The delay-locked loop aligns the outcoming edge with the incoming edge by regulating the current that drives the 64 delay line stages. A static error of 60 ps of this alignment is expected from the simulation for a typical NMOS and PMOS device corner. Each of these subdivisions can be tapped for controlling cycle-related elements. These cycle-related elements are (i) the start and (ii) the end of the laser pulse, (iii) the corner points of the triangle waves, and (iv) the position (start and stop signals) of the (optional) SPAD gating of the pixels (not reported here but used in [24]).

The laser driver (EPC21603) is off-chip and driven by an LVDS output to avoid supply bounce correlated with the laser pulse. The VCSEL array used in the experiments is from OSRAM-AMS (EGA2000-850-N). A 16 mm/F1.6 VIS-NIR lens (Edmund Optics #67-714) was used with a bandpass filter (850 nm center, 50 nm FWHM). The angular horizontal field of view (FoV) for the 9.6 mm horizontal sensor width and the used lens is 33°. However, the maximum horizontal FoV for the lens is around 31°, limiting the maximum possible FoV of the sensor. The second stage average switching rate, f_s_, which may be varied over time (e.g., 800 kHz and 80 kHz), is interrupt-driven by the FX3 microcontroller, and its propagation (F3, F4) over the full array is performed by the image sensor circuitry in a rolling wave mode, i.e., from row to row, to spread the current drawn from the power supply. During subframe readout periods, f_s_ is halted to avoid clock feedthrough in the output values. The FX3 controller also controls when the photon counters are reset, when the gate is actuated, when readout happens, when the laser is enabled, etc.

The TSIN, TCOS, and Gate signals are array-wide signals that need to arrive everywhere at the same moment in time, which is a challenge. Each of these three signals have their own digital *tree* buffers, like in a *clock tree* that is known in digital chip design. An original signal drives two buffers, localized above the pixel area, at a quarter and three quarters of the full width of the sensing area. These two buffers then each buffer two (or four) other buffers, equally spread over their half of the sensing area. This happens several times until per column there is one buffer to drive the column line (for the Gate signal). Attention is given so that the capacitive loads of the time-critical nodes are such that all buffers of the same level switch at the same moment in time. Also, local power supply decoupling is foreseen for avoiding local drops in supply voltage. For the TSIN and TCOS waves at the end, the digital outputs drive the current sources alternatingly up and down on the TSIN and TCOS nodes. Two small loops regulate the current source and sinking amplitudes so that the target top voltage and bottom voltages are reached in a synchronous and stable way.

**Figure 10:**
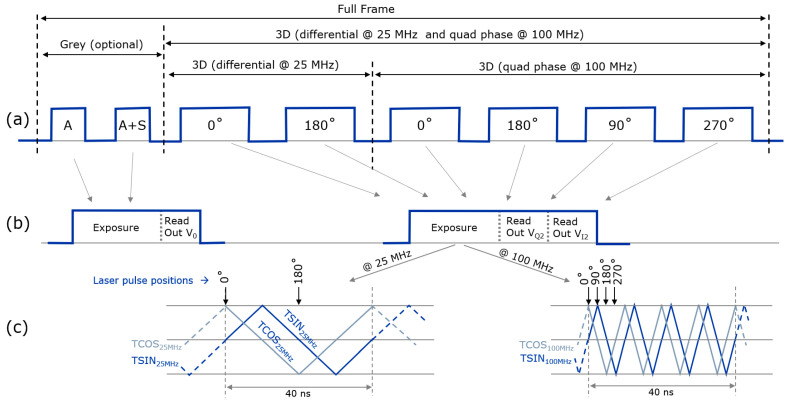
Timing diagram containing (**a**) subframes for gray value and 3D acquisition; (**b**) exposure and readout subframes, and (**c**) demodulation signals for 25 and 100 MHz subframes with the laser pulse position depending on the phase of the frame.

The system works with subframes (Figure 10) for capturing gray values and 3D subframes. Only photons with a wavelength close to 850 nm are captured due to the presence of the optical bandpass filter. The photon counter can thus only be used to make frames based on this wavelength. Gray subframes with ambient light only, and with both ambient light and a pulsing laser, can be made. By subtraction, one can then construct an estimation of the laser light intensity without ambient light. The exposure times for these gray captures are typically much shorter (between 100 ms and 1 ms) than the ones used for 3D to avoid photon counter saturation.

Many subframe configurations can be used for 3D acquisition; the ones used in Section 5.1 and Section 5.2 are demonstrated by Figure 10b. In the differential acquisition, two subframes are recorded: one with the laser pulse at the start of each cycle (0°) and the second with a 20 ns delay (180°). In the six-subframe approach (Section 5.2), two subframes are made at 25 MHz (with phases 0°, 180°) and four at 100 MHz (with phases 0°, 180°, 90°, 270°), as indicated.

## 5. Operation of the CA-dToF Camera

In this section, we show various operational modes, starting with a differential operation, followed by a *dual frequency* approach without and with spatial filter application at the PC level. Two additional experiments show that the system does not saturate at longer exposure times, and that it can handle a large range of A and S photons within the same image. A first attempt to evaluate a higher A operation with a moving wall in various ambient light conditions is also included.

### 5.1. Differential Operation Using (0°, 180°) Phases at 25 MHz

**Figure 11:**
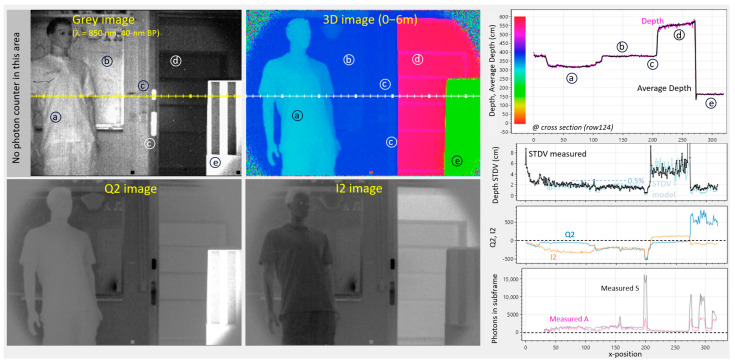
Demodulation using (0°, 180°) phases at 25 MHz: Gray, Q2, I2, and 3D images. Shown right are cross-sections from the image’s row 124, giving more quantitative results, including measured and modeled depth STDV. Color scale is present in the depth-graph (upper right). Ambient is 1 klux at the whiteboard (b) and 2 klux at the box (e).

The setup of the following measurements is very simple: there is a camera, like in Figure 9, upper left, having one VCSEL array for the illumination of the scene. Ambient light comes from behind the camera through office windows, illuminating the scene with uncorrelated ambient light. Besides that, there is the scene itself, with several challenges as indicated in Figure 11: (a) is a mannequin, (b) is a whiteboard, (c) are retro reflectors for testing the dynamic range, (d) is a cabinet in the corridor, and (e) is a box with black and white stripes for testing color dependency.

In the subsequent demonstrations, we make use of (quite long) 20 ms exposure subframes to compensate for the low PDE. Figure 11 demonstrates the operation at 25 MHz demodulation, giving a 6 m unambiguous range. During each subframe, a VCSEL light transmitter, positioned close to the lens, pulses floodlight to the scene. Two subframes are recorded (0°, 180°), performing a differential measurement to cancel offsets from the source followers and column ADCs. The distance is calculated using the following equations:

(15) $$D=\frac{\Gamma }{4}\;\left[1+\frac{\left(\;V_{I2}^{180}-\;V_{I2}^{0}\right)}{\left|V_{Q2}^{0}-\;V_{Q2}^{180}\right|+\;\left|V_{I2}^{0}-\;V_{I2}^{180}\right|}\right]\;\;when\;\left(V_{Q2}^{0}-\;V_{Q2}^{180}\right)>0,$$

(16) $$D=\frac{\Gamma }{4}\;\left[3+\frac{\left(\;V_{I2}^{0}-V_{I2}^{180}\right)}{\left|V_{Q2}^{0}-V_{Q2}^{180}\right|+\left|V_{I2}^{0}-V_{I2}^{180}\right|}\right]\;\;when\;\left(V_{Q2}^{0}-V_{Q2}^{180}\right)<0,$$

whereby the common voltage is conveniently eliminated. Since two subframes are used, $n_{sf}=2$ . Only the position of the laser is changed (0°, 180°) between the two frames, and thus the measurement depends only on small signal differences linked to the difference in laser pulse position. This makes it very robust and independent of absolute values. For example, the vertical position and the amplitude of the TSIN and TCOS waves become relatively unimportant. We can set them to oscillate between 100 mV and 900 mV, but also, between 200 mV and 600 mV, yielding the same depth estimates of the objects in the scene.

In the gray image, the first 32 columns have been reserved for some special experiments not considered here. This image is taken with and without the laser enabled, in that way providing a method for deducing A and S. Choosing the gray image exposure period manually allows us to avoid saturation whilst still having enough photons to give a somewhat reliable number for the darker scenes. Taking into account the exposure period ratios (of the gray subframe and of the depth subframes), an accurate estimate for the depth-subframe’s A and S number of photons can be extrapolated (assuming a fixed laser output amplitude).

The recorded Q2 and I2 images (Figure 11 bottom) give a mix of gray and phase information. However, when used in accordance with Equations (15) and (16), they deliver a depth map in the 0–6 m range, effectively canceling the color dependence (gray value).

The magenta curve on Figure 11, upper right, is the estimated distance for each frame, whilst the black curve is an average over 100 measurements for measuring the accuracy and for the calculation of the distance STDV. The modeled STDV, based on Equation (14) and on the extrapolated A and S, can now be compared with the measured STDV. The results show a good agreement, and a relatively good precision of 0.5% is observed at the center of the image. There is no depth color dependency noticeable at position (e).

The retro reflector gives a 15-times increase in signal photons and a 4-times increase in ambient photons (at column 200). The depth estimate is not affected; however, improvements in the modeled and measured STDVs are clearly noticeable.

### 5.2. Dual Frequency: (0°, 180°) Phases at 25 MHz and (0°, 180°, 90°, 270°) Phases at 100 MHz

**Figure 12:**
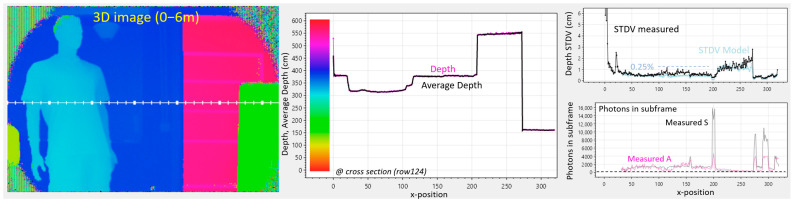
Demodulation at (0°, 180°) phases at 25 MHz and (0°, 180°, 90°, 270°) phases at 100 MHz. Ambient situation is the same as the previous experiments. Color scale is present in the depth-graph (center).

In Figure 12, a rough initial distance estimate is made at 25 MHz (with 0° and 180°) (similar to Section 5.1), followed by a much more precise 4-phased estimate at 100 MHz, similar to [4] in iToF. T_cycle_ remains at 40 ns; the laser remains pulsed at the same 40 ns intervals; however, the triangular wave generators are directed to oscillate four times per cycle, based on the four-times more corners provided from the 64 divisions (Figure 10). The second measurement now has a four-times reduced unambiguous range of Γ = 1.5 m, and an $n_{sf}=4$ (from the four phases). The 6 m range is thus combined with a much-improved precision (4 × $\;\sqrt{2}\;$ better in theory) and accuracy, at the expense of a 3-times lower framerate.

### 5.3. Nearest Neighbor Spatial Filtering Operation

**Figure 13:**
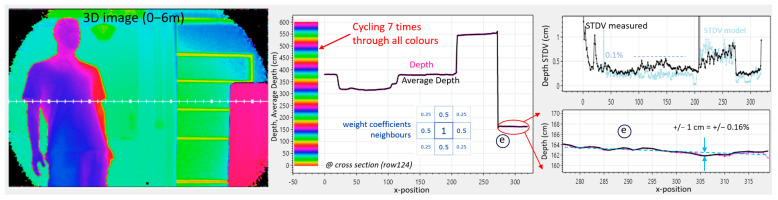
Demodulation at (0°, 180°) phases at 25 MHz and (0°, 180°, 90°, 270°) phases at 100 MHz, *including* a spatial filter in post-processing. Same ambient situation as in Figure 5. Fixed pixel noise (in cm) is demonstrated on the “flat” surface of the box (e). Color scale is present in the depth-graph (center).

In Figure 13, the camera conditions from Figure 12 are reused, but the outcome is followed by spatial filtering in post-processing on the VQ2 and VI2 using 50% of the pixel’s nearest neighbors and 25% of its diagonal neighbors.

Since the distance is now based on a 4-fold number of measurements compared to the previous example ( $totalling{\;n}_{sf}=16$ ), the predicted STDV is further reduced by a factor of two. Also, the measured STDV was reduced by two, but an unknown issue was preventing the STDV from reducing below 4 mm.

The measured STDV is, however, quite good percentagewise (0.1%). Another way to visually recognize this, is that, *even with the color scale cycled seven times over the 6 m range*, little noise in the 3D depth picture is visible (left).

It is important to consider that spatial filtering can be performed either at the (I, Q) level or at the distance level. By conducting it at the (I, Q) level, a pixel with less amplitude automatically reduces its own weight. This was confirmed for a pixel with a very high DCR (a so-called screamer): its low Q and I effortlessly reduced its weight through averaging. This seamless weighting will not occur should *the distances* be averaged.

A drawback of spatial filtering is that, on sharp edges, the effective spatial resolution will be somewhat reduced. But the fact that accuracy and precision are improved significantly makes it worth implementing. Using multiple pixels and multiple phases in the evaluation of the distance leads to accuracy improvements and reduces variability on every level of the analog signal chain. An accuracy detail is on the bottom right of Figure 13: the distance to box (e) with the white and black stripes appears flat, as it should: the measurement deviates from a straight line by only +/− 1 cm (+/− 0.16%). Note that this is accomplished *without any calibration*, neither at the pixel nor the column level.

### 5.4. Evaluation of Cyclic Errors

**Figure 14:**
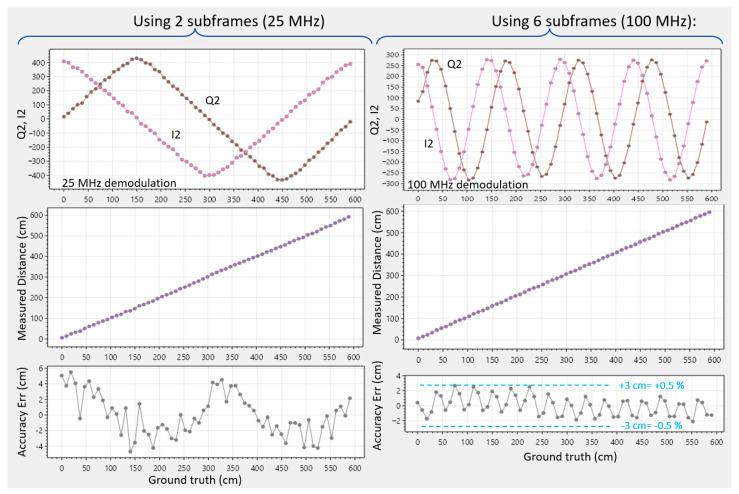
Accuracy of a pixel in the center of the image. The laser is cycled over the full 360° phase range in 64 steps. The level of cyclic error is then obtained by plotting the accuracy error, being the difference between the calculated distance (derived from Q2 and I2) and the ground truth.

To investigate the error generated by various sources on the distance measurement, we have made a test procedure that can easily measure accuracy errors over phase variation, as shown in Figure 14. One of the specific purposes was to check the effect of the non-linearity of the used averaging capacitances, as shown in Figure 3.

Any pixel in the image can be selected, having its particular A, S, and distance conditions. We cycle the laser pulse through the 64 positions and measure Q2 and I2 for each phase and plot the associated distance. Through this demodulation, we sample the triangle waves at the given ASR condition. In each triangle corner, there will be some rounding due to convolution with the laser pulse width.

In our experiments, the laser pulses were ~2 ns FWHM. Using Equations (15) and (16), we obtain a distance between 0 and 6 m. The deviation from the ground truth is the accuracy error, revealing limitations in the depth estimation principle. Measuring the ground truth versus distance in real circumstances (as is performed in Section 5.7) takes more time and requires a specific setup, whereby the effect of phase variation and signal S intensity variations are varied simultaneously. S reduces strongly with the square of the distance. In that way, a mix of effects is recorded, which is unfortunate because, when an error occurs, it cannot be uniquely attributed to the phase or S variation. The advantage of only cycling the laser pulse position is that cyclic errors due to phase errors can now very quickly be uncovered independently of the S variations.

Figure 14 (left) shows the cyclic error with a differential measurement. Q2 and I2 show slightly rounded triangle corners due to a convolution with the 2 ns laser pulse. This is with a pixel at 370 cm depth, A and S at ~1000 photons per subframe, and ~1 klux of ambient light. When cycling through the various laser positions, additional averaging is applied in post-processing to study the accuracy with reduced temporal noise. The error is +/− 6 cm, or +/− 1%. On the right, the case of dual frequency with spatial filtering is tested, revealing the 100 MHz demodulated triangle waves, the derived distance, and its accuracy error. The dual frequency approach helps to improve accuracy to +/− 0.5%. Cyclic errors have 16 oscillations due to the increased frequency. Notably, the demodulated waves have become more sine-like due to the relative increase in importance of the 2 ns laser pulse with respect to the shorter 10 ns triangle period. This effect was aggravated experimentally by lengthening the laser pulse, confirming the assumption. The application of a shorter laser pulse, on the other hand, was not possible with the used laser driver/VCSEL combination.

Interestingly, a few pixels in the array expressed increased cyclic error in the differential measurement. This was attributed to a difference in amplitude between the Q2 and I2 of the pixel involved (for example, due to different PMOS transistors used in the voltage followers). Fortunately, with 4-phased measurements (either with dual or with a single frequency), due to the combinations of the various sub-measurements, the aggregated amplitudes become the same by construction, and hence this source of cyclic phase error is automatically largely compressed. For improving accuracy, the 4-phased operation is thus helpful. Also, these experiments show that the non-linear capacitive behaviors (Figure 3 and Section 3.3.3) can only have very little impact on accuracy.

### 5.5. Exposure Time Freedom

Whilst experimenting with the camera under various light conditions, the exposure time for the gray-imaging photon counter required careful setting to avoid image saturation. Conversely, the true benefits of the CA-dToF are highlighted by its non-saturation behavior, since the averaging process never saturates I2 and Q2. Consequently, one is free to choose an exposure time and set the toggling rate(s) in accordance with the principle explained in Section 3.1.

**Figure 15:**
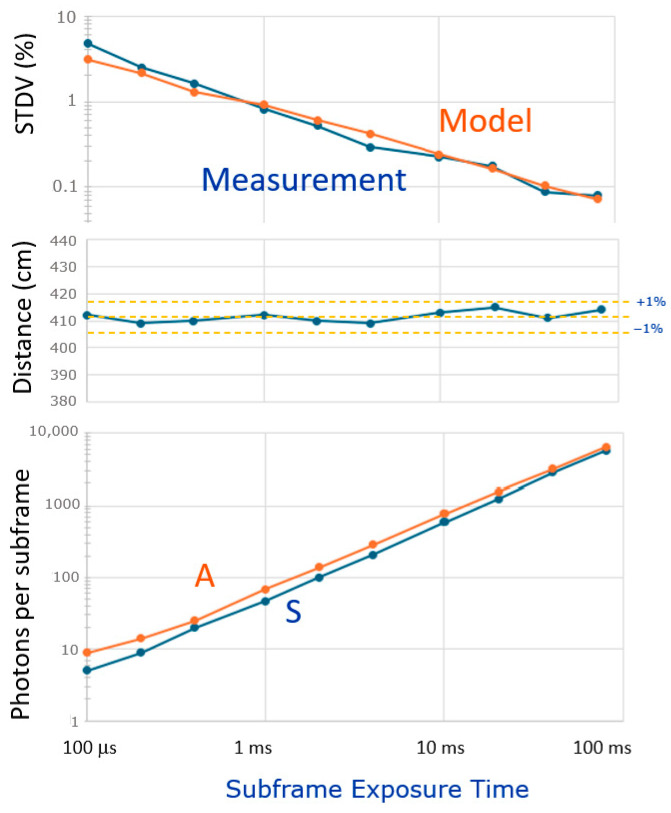
Experiment showing variable exposure time for depth sensing, from 100 us to 80 ms. The measured average A and S (**bottom**) are indicated, as well as the measured and modeled STDV, based on A and S (**top**). Depth variations remain within +/− 1% (**middle**).

To demonstrate this resilience, we selected a pixel at a distance of 410 cm and under an illumination condition of A ≈ S at 1 klux ambient light. Differential measurements were performed with a single frequency (25 MHz), with the spatial frequency filter applied ( $n_{sf}=8$ ) and the exposure time for the depth measurement being varied over *nearly 3 orders of magnitude*: from 100 ms to 80 ms (limited by a software bug). The accuracy was not impacted (+/− 1%). These results are shown in Figure 15. During the measurements, the ambient weather conditions slightly changed, and the ambient light was almost twice as much during the short exposure times. Nevertheless, the distance remained at 410 cm (+/− 1%) over the various exposure conditions. The measured standard deviation is well in accordance with the modeled one. The shortest exposure time is an interesting case: per subframe, each pixel receives only S = 5 and A = 10 photons. Still, an STDV of 5% is reached after spatially filtering (as in Figure 13).

This demonstrates *one of the true benefits* of CA-dToF. By not binning the incident photons (and thus not confusing their ToAs), by operating shot-noise limited, and effectively applying the CoM on the received signal photons, a system is created that starts giving reasonable depth estimates from a low number of signal photons S onwards.

### 5.6. Use Under Higher Ambient Light Conditions

**Figure 16:**
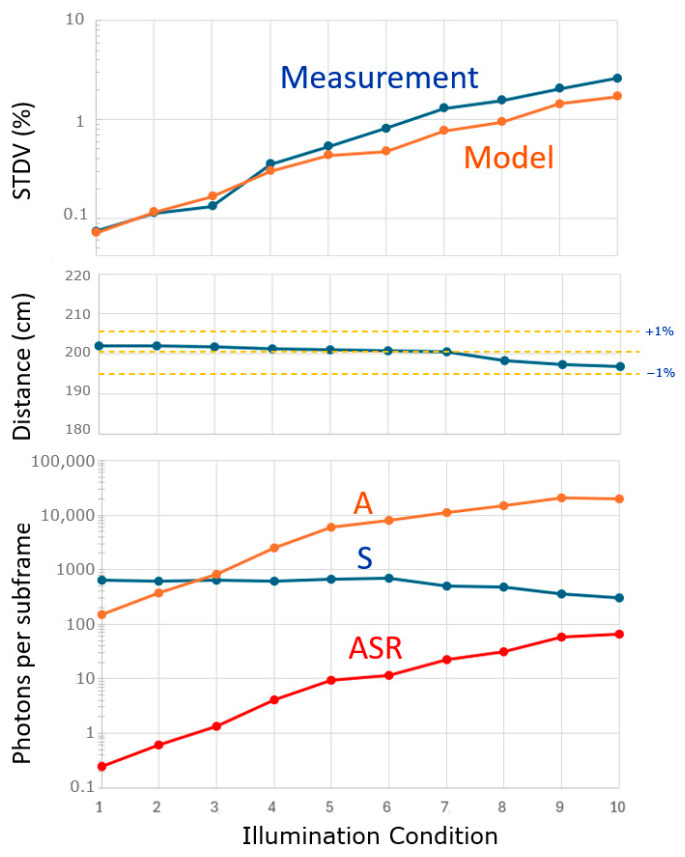
Experiment with an increasing amount of ambient light from A = 251 to 20,066 photons per subframe (**bottom**). Indicated are A, S, and ASR (**bottom**). The measured distance (**center**) and the modeled and measured STDV (**top**).

Another experiment worth performing is to vary the ambient light over a large range, keeping all other conditions the same, including the object distance. To study this, we selected a pixel at a distance of 200 cm. The light of a dimmable halogen spot was focused on the area of interest, and, with 10 different ambient conditions (varying A), the curves of Figure 16 were recorded. Four-phased measurements were conducted at a single frequency (25 MHz); the spatial frequency filter was applied ( $n_{sf}=16$ ), and the exposure time for the depth measurement was fixed at 2 ms. The lowest ambient condition was A = 251 and the highest was 20,066. The applied laser signal was fixed. Note, however, that the *measured value* decreased from S = 630 (when having low ambient conditions) down to 303 (at high ambient conditions). Together, the ASR ranged from 0.25 to 66.2. The changes in measured distance stayed within +/− 1% (middle graph); however, a tendency is observed that, for higher ambient conditions, the object appears to be closer. The standard deviation increased with more ambient light, as expected.

The STDV model still conforms; however, the measured outcome is somewhat worse at higher levels of ambient light. The sum (A + S) remained well below the AA = 8 × 10^4^ for all conditions. Since the ASR reached relatively high values, the ADC was set to the 11-bit resolution needed to achieve enough resolution at the lower output voltage levels reached during this measurement.

Measurements discussion: The fact that S reduces with increasing A could be attributed to the deadtime of the SPAD. Furthermore, the object appearing closer at higher ambient conditions can be attributed to the mentioned shadowing effect [22]: signal events prevent ambient events in their deadtime, and, therefore, the ambient events are no longer evenly distributed. Nevertheless, this demonstrates *the power of averaging*: even though the ambient light is 66-times stronger than the reflected laser light, it does not significantly affect the measured distance.

### 5.7. Moving Wall Experiment

**Figure 17:**
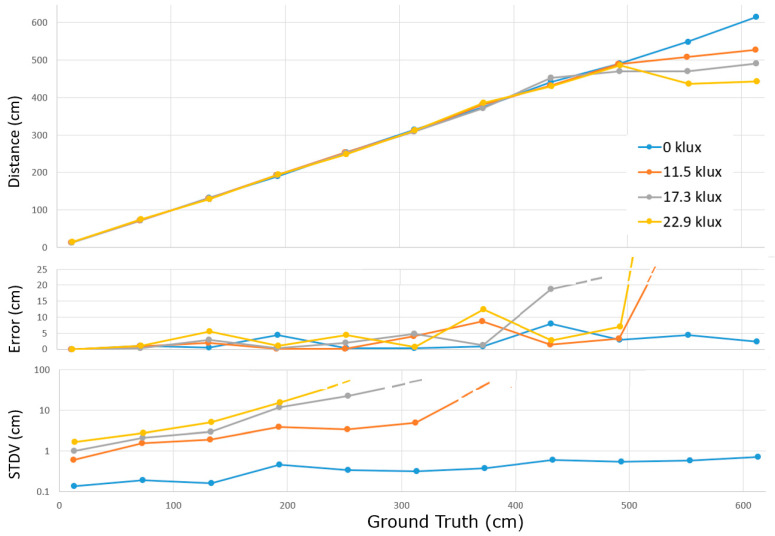
Estimated distance versus ground truth (**top**). Error on the distance (**center**); STDV of the estimated distance (**bottom**).

A ground truth experiment was performed under four ambient light conditions, as shown in Figure 17. Considering the low PDE, an additional VCSEL array was built-n, illuminating the scene with an average light power of 580 mW at 850 nm. Calibrated artificial sunlight was projected on a wall that could be positioned in the full unambiguous distance range. Four levels of ambient light (0, 11.5, 17.3, and 22.9 klux) were selected for the experiment. The subframe exposure time was 25 ms; the triangle frequency was 24 MHz and 96 MHz (for six phases), and the spatial filtering was applied. Using switches, a subsequent *third averaging stage* was included in the circuit, in principle allowing the averaging of 200 × 200 × 200 = 8 × 10^6^ samples. The toggle frequencies of the second and third stage were set at 80 kHz and 0.8 kHz. Since the experiment was performed in a limited time frame, performative conclusions should not be made yet.

Nevertheless, some observations can already be made. Since the averaging cannot saturate, exceptionally short distances can still be measured correctly (down to ~7 cm). At longer distances, due to the poor SPAD’s PDE and the quadratically reducing number of signal photons S for longer distances, the STDV gets out of hand (by several percent and then 10s of percent), which triggers two failure modes leading to large accuracy errors (>4 m). The first failure mode is that, when being close to the end of the unambiguous range, the phase wrapping suddenly gives a very short distance, which is detrimental for the average distance calculation. Furthermore, when the low frequency measurement delivers a distance error (in one of its measurements) of more than 75 cm, the algorithm picking the distance out of 4 may pick the wrong one, which then yields an error of 1.5 m, also jeopardizing the average distance calculation. When the standard deviation is high in the base frequency, it may thus be a safe choice to stick to the single frequency approach. Additional experiments need to be conducted on higher ambient light levels with moving walls, using additional laser light, before more final conclusions can be drawn on the performance of the present demonstrator in higher ambient light situations.

### 5.8. Additional Performance Parameters

The subframe rate with a 20 ms depth exposure was 24 fps. The differential frame rate was thus 12 fps for Figure 11, and the systems with dual frequency and six subframes were 4 fps (Figure 12 and Figure 13). Besides the long exposure time, the dataflow and many other time-consuming elements can still be optimized/parallelized. The column ADCs were running at 100 MHz with a 10-bit resolution, except for in the moving wall experiment, where an 11-bit resolution was used.

Concerning the image sensor power dissipation, in the experiments of Figure 11, Figure 12 and Figure 13, the supply for the SPADs needed to deliver 1 mA at 22 V in the case of 1–2 klux ambient light in the frame, with excess bias set at 2.4 V. Other than the SPAD voltage, the image sensor requires a single supply voltage of 1.8 V (Vdd). The current consumption of Vdd was 61 mA when recording differential frames, and 98 mA when recording at a dual frequency with six subframes. The higher current is due to the elevated frequency of the TSIN and TCOS triangle waves. Since the generation of the triangle waves is performed by simple current sources and sinks, a comparable power dissipation is reached, as when driving a full-swing digital signal over the array. Since the amplitude of these triangle waves is only half of the supply voltage, while the current is provided from the full Vdd voltage, one can state that providing a single clock signal to the full array at a full supply voltage consumes about the same amount as driving two triangle waves to half the supply voltage. Additionally, there is the power consumption for the generation of the non-overlapping clocks; however, the firing of the SPAD itself is consuming much more energy due to its relatively large capacitance, and the potential drop for the charges being over the 22 V SPAD supply instead of over the 1.8 V Vdd supply. The power dissipation needed by the second stage filter, which requires the clocks (F3, F4) to be switched over the full array, can be considered negligible, because it runs at a much lower frequency than the triangle waves.

## 6. Discussion

CA-dToF, as presented in this paper, operates with a short light pulse that is sent to the scene, whereby the received reflected photons are correlated with two orthogonal waves, precisely registering the ToA. The fact that it works (and can only work) with a short light pulse makes it thus a *direct time-of-flight system*. The returned short light pulse is thus registered upon arrival. This is in contrast with iToF, whereby the emitted light that traverses the scene is an amplitude wave, which is demodulated after reception by directing each photo-generated electron to one or another (floating) diffusion based on a demodulating signal.

However, since there are also *demodulation waves* involved, several commonalities with iToF [4] can be expected. Let us thus consider the *challenges that occur in iToF* and how they relate to CA-dToF:

- One of the challenges in iToF is the *cyclic error* that occurs when the shape of the modulated laser amplitude wave deviates from the targeted one (typically square or sine wave). This directly translates into a cyclic error behavior and possibly needs calibration. In the case of CA-dToF, the laser pulse is short, and its shape is less relevant. What counts is the CoM of the laser pulse, which, if it changes, leads to a global fixed distance error, not a cyclic error. Additionally, a deviation in the shape of the triangle wave leads to cyclic error. Fortunately, making a controlled electrical shape at 100 MHz is much easier than making a controlled optical wave shape.
- Another element is that most iToF systems use dual-tap receivers and need to perform the demodulation for the 0° and 90° phase in a separate subframe. As a result, laser-light shot noise is not canceled. In the case of CA-dToF, since both ASIN and ACOS waves are sampled simultaneously, laser-light shot noise is canceled (Section 3.2.2).
- Furthermore, in iToF, the ToAs are binned, and therefore precious information on the exact ToA is lost, which will lead to an increase in the distance’s STDV. CA-dToF can handle low-light levels quite well due to this, but also due to the fact that the operation is shot-noise-limited: at a level where iToF has not yet surpassed its *readout noise level*, a decent distance estimation can already be provided with CA-dToF.
- In iToF, one *integrates* charges generated by A and S photons, and one has to choose an exposure period *long* enough in order to accumulate *enough* signal to be above the readout noise, but also not *too long*, to avoid reaching the full well capacity. And this is for *all received illumination conditions (variables A and S) throughout the image*. Often, several different exposures need to be performed to obtain a full image coverage. At the edges of a different exposure, stitching distances leads to accuracy errors at the stitch boundary. CA-dToF cannot saturate over large ranges of A, S, and exposure times; this is a clear advantage. No calibration, except for the array’s general distance offset, is needed: this concerns a single delay value for the full array. In addition, 4-phase operation, together with the proposed spatial averaging, suffices to largely reduce variabilities, offsets, and non-linearities.
- An advantage of the iToF system is that it provides a gray image as well, which is required in most applications. In CA-dToF, a dedicated photon counter needs to be added in the circuitry for gray scale acquisition.
- It is an option in CA-dToF to use the fast-gating system to make the pixels blind for light-pulse reflections during (for example) the first 5% of T_cycle_: this can solve the multipath crosstalk induced by a common cover glass. Some of the nearby camera depth range will, however, be sacrificed.

Ohain the dToF side, several additional considerations can be made:

- dToF is implemented in a complex digital way and requires quite a large circuit, limiting the pixel pitch. With CA-dToF, a full resolution, low pitch, and low power may be in reach.
- Clustering multiple SPADs per pixel can be performed in CA-dToF. In dToF, this leads to a system deadtime that limits depth accuracy by blocking ToA registrations of clustered SPADs firing simultaneously. However, in CA-dToF, by giving each SPAD in the cluster its own non-overlapping clock circuit (Section 3.3.2), this can be avoided.
- The binning of photons (dToF) restricts the depth estimations when having low numbers of signal photons. CA-dToF uses each ToA to its fullest potential, resulting in an optimal CoM situation.
- On the other hand, the noise from ambient photons in the histogramming approach stems from the ambient photons in the histogram bins in and around the observed peak. This helps dToF long-range LIDAR systems operate in full sunlight.
- Multipath is not a source of concern in dToF when leading to multiple separable peaks in the histogram.

In Table 1, a short comparison is given of the representative (non-scanning) TOF systems. Ref. [25] is a SPAD-based dToF system, where clustered SPADs drive a histogram’s memory. This particular system has also the benefit of being integrated into a miniature package including VCSEL illuminators. Ref. [26] is another SPAD example that is this time iToF-based, with one SPAD per pixel, and whereby very linear analog photon counters are chopped to achieve a very good background light resilience. Ref. [27] is a more classical approach to iToF, whereby demodulation happens inside the detector device based on applied fields. Due to the small pixel pitch, this type of sensor allows for the highest resolutions.

## 7. Future Work and Conclusions

All the performance parameters are subject to much improvement when increasing the PDE from 0.7% to 40%: this can be achieved with an optimized image sensor design using 3D stacking technology. Sub-millisecond exposures can then be reached, frame rates increased, ambient light resilience improved, and power dissipation reduced.

This opens the way for high-resolution CA-dToF depth cameras expected to outperform iToF cameras in several ways. A high dynamic range with respect to both ambient and signal light can then be anticipated. A short-distance range can be included, and/or cheaper illumination systems can be used in certain applications. Increasing the laser illumination light levels can bring an outdoor range into reality, with a distance range determined by the applied laser light level.

## Figures and Tables

**Table 1:** Comparison with typical state-of-the-art TOF systems.

	This Work (IISW 2025) Kuijk	IEEE Sensors 2025 Martin [25]	ISSCC 2025 Choi [26]	IEEE JSSC 2018 Kato [27]
**detector**	SPAD	SPAD	SPAD	Demodulating Detector
**dToF/iToF**	dToF	dToF	iToF	iToF
**data treatment**	correlation assisted	histogram, clustered SPADs	analog counting	CIS
**pixel configuration**	320 × 240 = 76.8 k	*not available*	64 × 64 = 4096	320 × 240 = 76.8 k
**TOF channels**	320 × 240 = 76.8 k	54 × 42 = 2268	64 × 64 = 4096	320 × 240 = 76.8 k
**pixel pitch**	30 µm	*not available*	32 µm	10 µm
**wavelength**	850 nm	940 nm	850 nm	850 nm
**PDE**	0.7%	*not available*	4.72%	responsivity = 0.34 A/W
**demodulation** **frequencies**	25 & 100 MHz	*not applicable*	3.125 MHz	100 MHz
**distance**	0.1–6 m	5 cm–9 m (indoor) 5 cm–6.5 m (40 klux)	76 m (max)	1.5 m (max)
**frame rate (fps)**	4	60	50	60
**STDV**	<1 cm (6 subframes)	+/− 4.5 cm (indoor) +/− 6.5 cm (40 klux)	6.4 cm	5.9 mm

## Data Availability

Data is contained within the article.
